# Reconstruction of the anterior cruciate ligament using the Press‐Fit‐Hybrid technique—A cohort study with an up to 10‐year follow‐up

**DOI:** 10.1002/jeo2.70257

**Published:** 2025-05-06

**Authors:** Niko Schauer, Martin Fischer, Rejane Golbach, David A. Groneberg, Fabian Holzgreve, Daniel Niederer, Daniela Ohlendorf, Adalbert Missalla

**Affiliations:** ^1^ Institute of Occupational Medicine, Social Medicine and Environmental Medicine Goethe University Frankfurt Frankfurt am Main Germany; ^2^ Institute of Biostatistics and Mathematical Modelling University Hospital Frankfurt Frankfurt am Main Germany; ^3^ Ortho‐Klinik Rhein‐Main Offenbach Germany

**Keywords:** ACL reconstruction, anterior cruciate ligament (ACL), arthroscopic surgery, hamstring graft, knee ligament injuries

## Abstract

**Purpose:**

To examine the long‐term results of the Press‐Fit‐Hybrid fixation technique in anterior cruciate ligament (ACL) reconstruction on self‐reported knee function, sport ability, return to sport (RTS) success, quality of life and re‐rupture rates.

**Methods:**

Adults with ACL rupture which was reconstructed between 2011 and 2013 using the Press‐Fit‐Hybrid fixation technique were included. Participants completed questionnaires before surgery and at 6‐month, 1‐year, 2‐year, 3‐year and 10‐year follow‐ups. Subjective knee function was self‐reported using the International Knee Documentation Committee (IKDC) and Lysholm scores. The level of sport activities was assessed using the Tegner Activity Scale (TAS), and quality of life was evaluated with the Knee injury and Osteoarthritis Outcome Score Quality of Life subscale (KOOS‐QoL). RTS success rates were categorised into return to participation, RTS and return to performance.

**Results:**

Participants (*N* = 135) (81 males, 54 females; aged 32.3 years [SD 11.7]) were included. Mean scores at 2, 3 and 10 years were for IKDC 87.6 (SD 10.5), 89.2 (SD 11.0) and 86.5 (SD 14.8), while for Lysholm 92.1 (SD 8.4), 93.3 (SD 7.8) and 90.6 (SD 12.3). The KOOS‐QoL averaged 80.4; 70.0% scored above 80.0. After 3 years, 73% returned to their pre‐injury TAS‐level; 52% still performed at the pre‐injury level after 10 years. Average return to participation was within 5.9 months. 96% returned to sport within 9.6 months on average. 72% returned to performance within a mean of 12.2 months. Re‐rupture rates were 2.85% in the first 3 years and 5% between 3 and 10 years post‐surgery.

**Conclusion:**

Press‐Fit‐Hybrid leads to low re‐rupture and high RTS rates, restoring knee functionality and improved quality of life. Preliminary results need validation in a randomised controlled trial.

**Level of Evidence:**

Level III.

**Abbreviations**
ACL: anterior cruciate ligament
IKDC: International Knee Documentation Committee
KOOS‐QoL: Knee injury and Osteoarthritis Outcome Score Quality of Life
RTS: return to sport
SD: standard deviation
TAS: Tegner Activity Scale

## INTRODUCTION

Surgical reconstructions of ruptured anterior cruciate ligaments (ACLs) mainly differ in terms of graft selection and fixation [34]. The interference screw is the most commonly used method of graft fixation but carries the risk of causing graft damage intraoperatively and damage resulting from screw misplacement [32]. To reduce these risks, hardware‐free methods of graft fixation have been developed [7, 13] and have shown good, long‐term results [1, 12, 14, 30, 39].

Press‐fit describes a category of hardware‐free fixation in which the graft is secured by placing bone plugs on either side to hold it in place within the femoral and/or tibial tunnels [32]. When compared to screwing techniques, the advantages of this fixation method include reduced post‐operative enlargement of the bone tunnel, direct bone‐to‐bone healing in the drilled channel, faster rehabilitation, avoidance of hardware‐associated complications and simpler interventions for revisions [16, 34].

Usually, quadriceps grafts are selected for the press‐fit approach [6, 27, 31]. More recently, Paessler and Mastrokalos [28] and Felmet [10] used the hamstring tendon as a new graft alternative for press‐fit fixation; the authors found comparable biomechanical stability and less donor morbidity than in the quadriceps techniques. Press‐Fit‐Hybrid utilises bone cylinders for the tibial and femoral fixation of the hamstring graft in addition to an extracortical button for further femoral graft fixation. Thus, this combines features of both close‐to‐the‐joint and distant‐to‐the‐joint fixation methods that may improve the postoperative outcomes for the patient. Given the extensive research already conducted on alternative ACL reconstruction techniques with long‐term follow‐up periods [12, 14, 39], this study aims to contribute to the existing body of findings on the new Press‐Fit‐Hybrid technique.

This study examines the outcomes of the Press‐Fit‐Hybrid fixation over a 10‐year follow‐up period to determine if it results in prolonged benefits and improved patient quality of life. The primary aim was to evaluate patient‐reported functional outcomes. As the incidence and outcome of ACL ruptures differ both between genders and different levels of sports activity [26], secondary aims were to identify between‐group differences (gender; level of sports activities), to analyse the process of returning to sports activities and to assess the rates of graft failure.

## MATERIALS AND METHODS

### Study design

This was a cohort study to investigate the outcomes of a fixation method for ACL reconstruction over an up to 10‐year follow‐up period.

**Figure 1:**
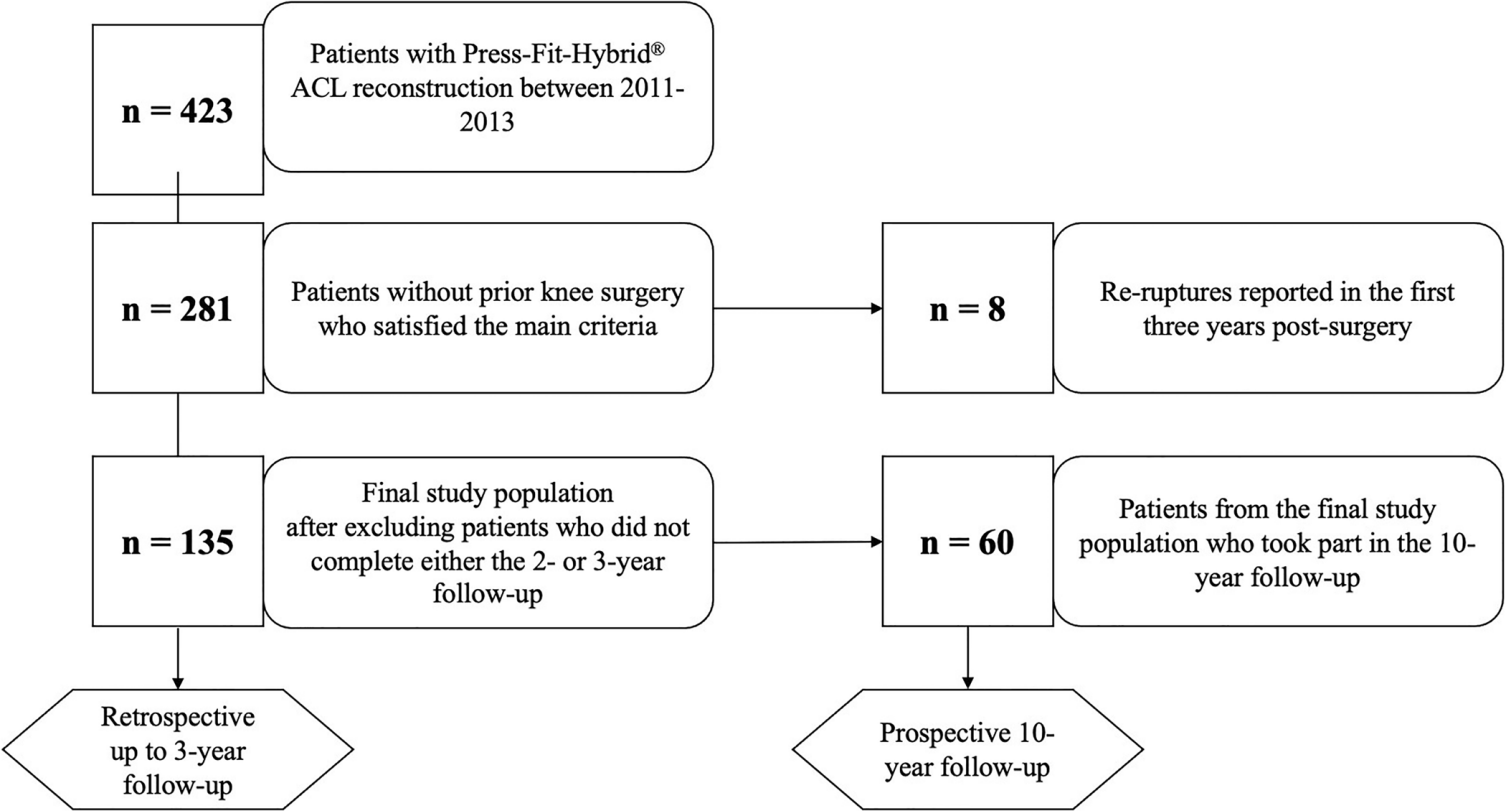
Flow chart of the study's participants. Numbers at each stage of the study are displayed. ACL, anterior cruciate ligament.

The study was based on surgical outcomes of a single surgeon who completed 423 ACL reconstructions between 2011 and 2013 (Figure 1).

The data were collected via surveys that were distributed to the participants in paper format as part of the patients' medical treatment and follow‐up visits during the first 3 years post‐surgery. For the 10‐year follow‐up, data collection and evaluation were conducted electronically via a digital survey tool in 2023. Participants were contacted by telephone, post and mail and could participate through a web link. The completed questionnaire was returned automatically by the programme.

### Participants

#### Inclusion criteria

Patients of the hospital between 2011 and 2013, of at least the age of 18 or, if younger, those whose knee growth plates had fused at the time of surgery, were included if they were undergoing an ACL reconstruction using the Press‐Fit‐Hybrid technique. Additional procedures to address meniscus and cartilage injuries during the intervention were deemed permissible, such as meniscal debridement.

##### Exclusion criteria

For patients who had undergone previous knee joint surgery, the ACL reconstruction was not the first surgery on the target knee. Patients who had sustained additional injuries to the lateral collateral ligament and the posterior cruciate ligament and patients who failed to complete either the 2‐ or 3‐year follow‐up were excluded. Patients who suffered an ACL re‐rupture during the first 3‐year follow‐up were excluded from subsequent follow‐up surveys and recorded as a re‐rupture in the statistical analysis.

### Surgical and therapeutic procedures

**Figure 2:**
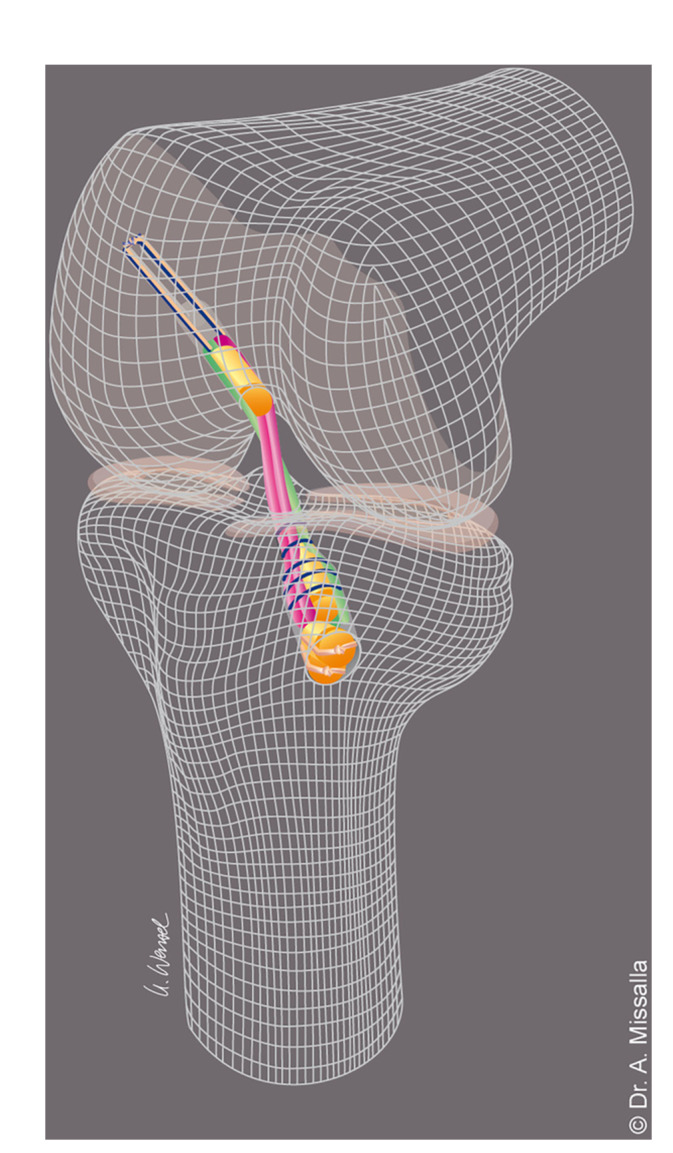
Schematic representation of the Press‐Fit‐Hybrid technique for ACL reconstruction. ACL, anterior cruciate ligament.

The ACL in all patients was reconstructed by the same surgeon using the standardised Press‐Fit‐Hybrid technique (Figure 2). To ensure reproducibility of the results, the surgery was performed using specialised instruments (developed by BIOMEDIX in collaboration with Dr Adalbert Missalla).

To harvest the hamstring graft, an incision was made over the pes anserinus and, depending on their thickness, either the semitendinosus or additionally the gracilis tendons were stripped. The further procedure was conducted arthroscopically, primarily through a high anterior lateral and deep anterior medial portal. The tibial tunnel was created using a single‐use hollow cutter (AlphaLock, BIOMEDIX), which produced an 8.24 mm diameter tunnel in under 3 s without causing overheating or bone necrosis. In addition, it provided a bone cylinder that was used for graft fixation. The correct positioning of the tunnel was ensured by an aiming device whose hook was placed on the tibial ACL stump at 90° flexion of the knee.

Based on the standardised diameter of the drilled tunnel of 8.24 mm, the Press‐Fit‐Factor (BIOMEDIX), an optimal relationship between the variable diameter of the implant (7.5–10 mm) and the implant bearing was developed to create the optimal fixation of the graft. Consequently, the tibial tunnel was prepared by several dilators to form the implant bearing. The dilatators required for each graft were determined by a scheme that respects the Press‐Fit‐Factor, according to the individual graft's diameter. The utilisation of asymmetric‐shaped dilators at the end of the procedure recreated the anatomical footprint shape and allowed for the reconstruction of the graft as a double‐bundle.

**Figure 3:**
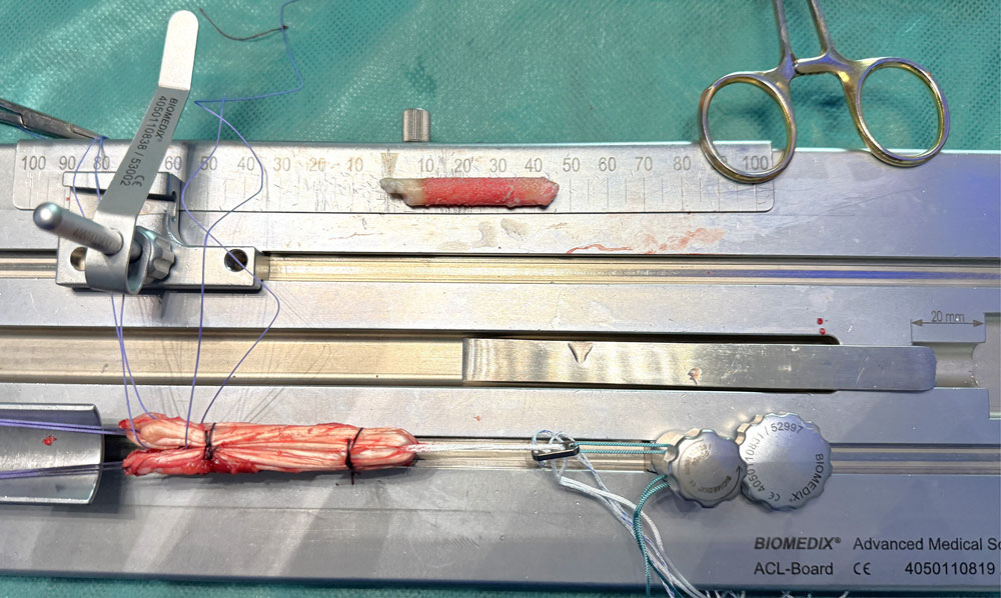
Preparation board (BIOMEDIX) with tibial bone cylinder and hamstring graft with its loop wrapped around the sling of an adjustable PullIUP® (SBM) fixation.

The graft was then placed onto a preparation board and folded to form a four‐ to eightfold bundle (Figure 3).

The loop of the graft was wrapped around the sling of an ultra‐button, with the tibial bone cylinder fixed between the loose ends of the tendon with stitches going through the bone and implanted to create the distal end of the graft. The final graft should have a thickness of 7.5–10 mm on the tendon segment.

**Figure 4:**
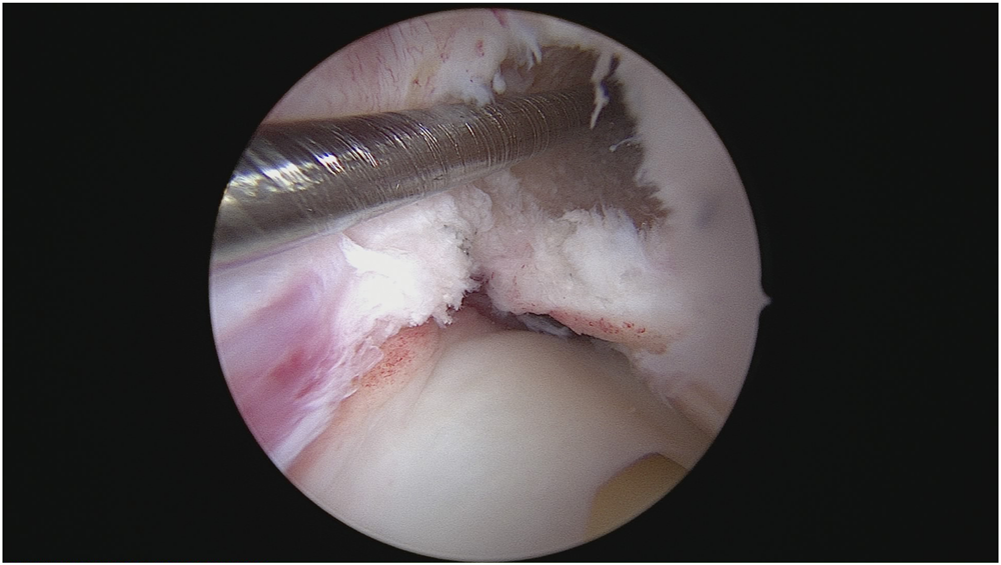
Femoral canal after removal of the bone cylinder.

The femoral tunnel was created with another single‐use hollow cutter (AlphaLock, BIOMEDIX) with a diameter of 8.24 mm and a depth of 25 mm at 130° flexion of the knee (Figure 4).

The aiming device was positioned on the lateral posterior edge of the notch. The bone cylinder thus created was extracted and the tunnel prepared by the corresponding dilators, as determined by the scheme based on the Press‐Fit‐Factor (BIOMEDIX). The lateral supracondylar femoral cortex was drilled with a button drill.

**Figure 5:**
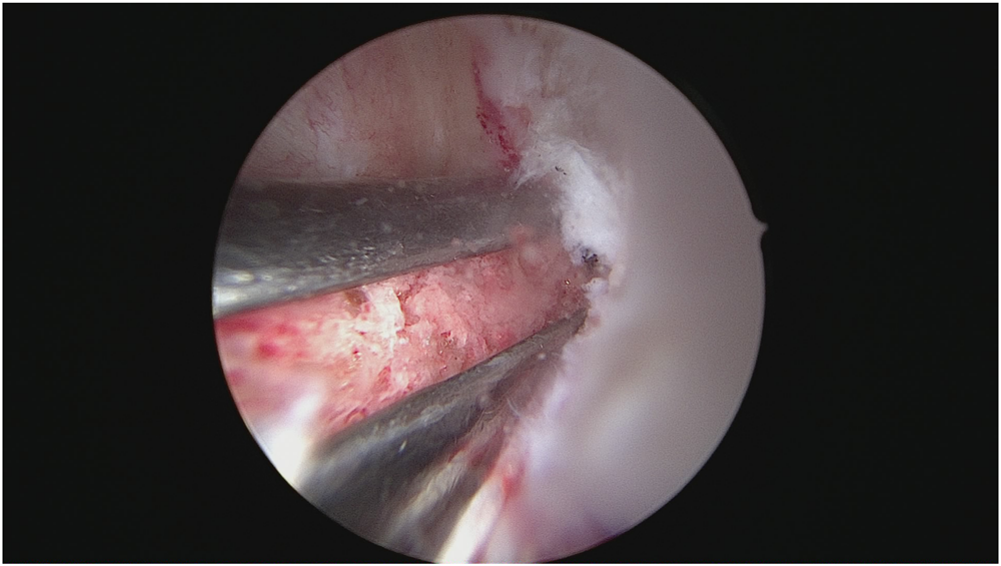
Fixation of the graft with the bone cylinder after insertion into the femoral canal.

**Figure 6:**
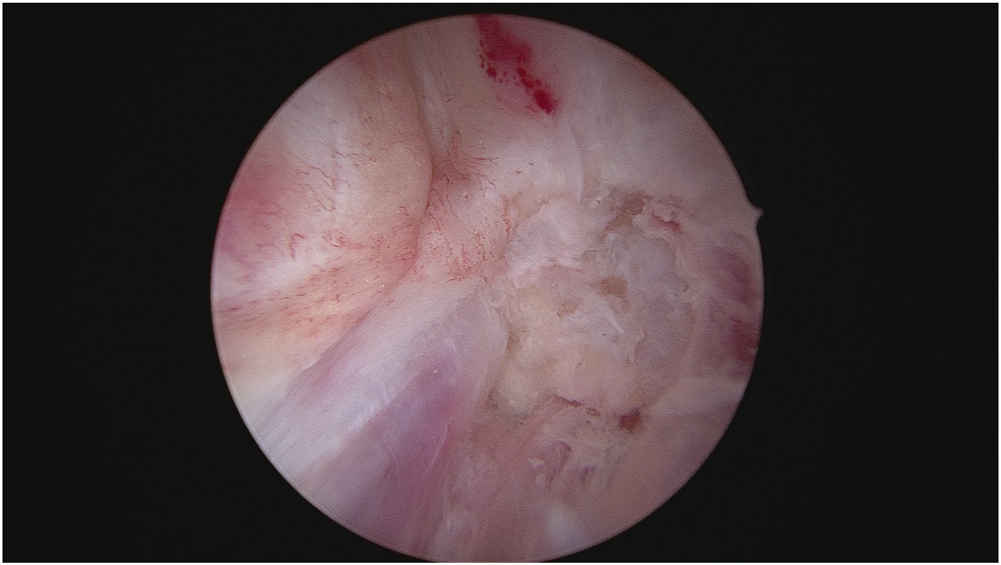
Femoral press‐fit fixation after application of the bone cylinder.

The graft was inserted in the distal to proximal direction at 130° flexion of the knee. The femoral side was pulled until the graft reached its predetermined position and was press‐fit fixated on the tibial end, close to the joint. The ultra‐button was tightened extracortically for additional femoral fixation distal to the joint. To secure the graft, the femoral bone cylinder was impacted into the bone tunnel adjacent to the graft, creating a press‐fit fixation close to the joint as a second femoral fixation (Figures 5 and 6).

As a second tibial fixation, the threads of the implant end were knotted to the bone. The arthroscopic surgery concluded with an examination of the graft's tension, positioning, and freedom from impingement, up to full extension.

All patients received identical postoperative treatment according to the guidelines of the German Society for Trauma Surgery [21]. Patients were instructed to wear a splint for the first 6 weeks after the surgery, allowing only 0°/0°/90° extension/flexion. During that period, they were further advised to train for full extension of the leg and physiotherapy was administered.

### Study procedures and outcomes

All patients completed questionnaires before the surgery and at five follow‐ups: 6 months, 1 year, 2 years, 3 years and 10 years post‐surgery.

To measure the knee's subjective constitution, function, and limitations, the International Knee Documentation Committee (IKDC) Subjective Knee Evaluation Form [15] was used. It ranges from 0 to 100 points, with scores >80 being defined as good or very good [15]. The IKDC is a reliable and valid tool for detecting functional changes after surgery [9].

The subjective outcome related to knee function in daily activities and knee laxity was assessed using the Lysholm score. It ranges from 0 to 100 points with results >84 being defined as good or very good [22, 36] and has been demonstrated to be a reliable and validated tool, being ideal for detecting the effects after undergoing an ACL reconstruction [8, 9].

As the patients performed a variety of sports at differing levels, the Tegner Activity Scale (TAS) [8, 36] was used to assess and quantify these activities and to facilitate their comparison. Furthermore, the changes in TAS over time can be used as an indicator of the medium and long‐term return to sport (RTS). Patients were categorised as non‐competitive athletes when reporting a score <7, or competitive athletes with a score ≥7 [36].

To evaluate the long‐term effects of the surgery on the patient's daily quality of life, the questionnaire used on the final follow‐up was supplemented by the knee‐related Quality of Life subscale of the Knee injury and Osteoarthritis Outcome Score (KOOS‐QoL). To analyse the process of returning to sporting activities, the questionnaire included questions regarding RTS [25, 35, 38]. The benchmarks for RTS were divided into three categories according to Ardern et al. [2]: return to participation, RTS and return to performance. Return to participation was defined as the athlete participating in rehabilitation and training. RTS was defined as the athlete performing his/her defined sport but not at pre‐injury performance level. Return to performance was defined as the athlete having returned to his/her defined sport at or above preinjury level.

As the study was conducted in Germany, the surveys utilised the German versions of the scores. These translations were shown to have similar psychometric properties to the original English versions [17, 18, 40].

### Re‐ruptures

Re‐ruptures are calculated as two rates by dividing the absolute numbers of registered re‐ruptures by the number of patients who were seen for follow‐ups.

The re‐rupture rate for the first 3 years post‐surgery was calculated based on all patients who met the primary inclusion criteria and were seen for clinical follow‐up (see Figure 1). Patients who reported a re‐rupture during this time underwent a physical examination and diagnostic imaging to verify the occurrence of the re‐rupture.

For the 10‐year follow‐up, patients were contacted and invited to complete an online survey; they were not seen in person. Therefore, re‐ruptures that occurred between 3 and 10 years post‐surgery were diagnosed based on the responses given in the questionnaire at the final follow‐up. Due to the high rate of patient loss between the 3‐year and 10‐year follow‐ups, a separate re‐rupture rate for the period between 3 and 10 years post‐surgery was calculated. This rate accounted only for patients who participated in the final follow‐up (see Figure 1).

As patients who suffered a re‐rupture or contralateral rupture between 3 and 10 years after the surgery were part of the first 3‐year follow‐up analysis, they were still included in the 10‐year follow‐up survey in order to analyse their development and present as much data as possible on the final follow‐up.

### Statistical analysis

The data were statistically evaluated using R Statistical Software (v4.3.2). Continuous variables were reported as means and standard deviations (SDs), non‐parametrical data as medians and ranges. Categorical variables were reported as absolute and percentage frequencies.

A non‐linear mixed‐effects regression, fitted by maximum likelihood (ML) was used to determine differences in IKDC levels over time between gender as well as level of sport activity. The relationship between IKDC and time (*t*) was assumed to follow the function IKDC=Asym+m⋅t+(R0−Asym)⋅exp(−exp(lrc)⋅t), where R0 denotes value of IKDC for *t* = 0, lrc denotes the rate constant of growth up to the maximum turning point, *m* denotes the decrease of IKDC after the maximum turning point, and Asym denotes the IKDC value for infinite time. Gender or sport activity were both modelled to have a fixed effect on the parameters Asym, R0 and *m*. Additionally, a constant random effect by patient was included in Asym and R0. The fit for parameter lrc was held constant for all influence factors and patients.

The likelihood of returning to performance was compared between competitive and non‐competitive athletes and between the genders using the log‐rank test. A *p* value of less than 0.05 was considered significant for all significance tests.

## RESULTS

### Participants

The number of patients at each stage of the study is shown in Figure 1. During the first 3 years, drop‐outs resulted from patients not returning the questionnaire or returning them incomplete. After 10 years, 73 patients could not be successfully contacted due to changes in phone number, address or email. Two refused to participate in the final follow‐up survey due to personal reasons.

#### Patient demographics

The patient demographic data are presented in Table 1.

**Table 1:** Patient demographics and characteristics of the study's participants.

Number of patients included	135
Age at the surgery in years (SD)^a^	32.3 (11.7)
Range (min.–max.)	13–60
Body mass index (SD)^a^	25.3 (4.3)
Range (min.–max.)	14.1–39.3
Male (% male)	81 (60%)
Female (% female)	54 (40%)
Right knee/left knee	80/55
Time between injury and surgery in months (interquartile range)^b^	2 (2/4.75)
Range (min.–max.)	2–264
Patients with time to surgery ≤ 3 months (%)	85 (63%)
Level of activity (TAS) pre‐injury (SD)^a^	6.3 (1.6)
Range (min.–max.)	3–9
Patients with TAS ≥ 7 (%)	69 (51%)

Abbreviation: TAS, Tegner Activity Scale.

a Values are given as a mean (standard deviation [SD]).

b Values are given as a median (upper/lower quartile limits).

#### Patient‐reported outcomes

Results of the self‐reported outcomes are shown in Table 2. Regarding the subjective knee function, 80.2% (IKDC) and 85.9% (Lysholm) of the patients showed good or very good results after 2 years, 81.1% (IKDC) and 85.7% (Lysholm) after 3 years and 75.0% (IKDC) and 80.0% (Lysholm) at the 10‐year follow‐up. The KOOS‐QoL yielded a mean score of 80.4, with 70.0% scoring above 80.0.

**Table 2:** Results of the patient‐reported outcome measures International Knee Documentation Committee (IKDC), Lysholm, Tegner Activity Scale (TAS) and Knee injury and Osteoarthritis Outcome Score Quality of Life (KOOS‐QoL) subscale.

			Pre‐injury	Pre‐surgery	6 months	1 year	2 years	3 years	10 years
Subjective knee function	IKDC score	Mean (SD)		56.8 (14.6)	76.2 (10.9)	84.0 (12.2)	87.6 (10.5)	89.2 (11.0)	86.5 (14.8)
		Valid questionnaires		87	98	116	81	37	60
	Lysholm score	Mean (SD)		63.1 (23.0)	85.9 (±11.2)	90.5 (9.1)	92.1 (8.4)	93.3 (7.8)	90.6 (12.3)
		Valid questionnaires		75	87	109	78	35	60
Level of sports activities	TAS	Median (range)	7 (3–9)	3 (0–8)	4 (1–9)	5 (1–10)	6 (3–10)	6 (3–10)	4 (2‐9)
		Performance at pre‐injury level (yes:no)			24%:8422%:78%	61%:5453%:47%	46%:3558%:42%	27%:1073%:27%	31%:2952%/48%
		Valid questionnaires	106	103	108	115	80	37	60
Quality of life	KOOS‐QoL	Mean (SD)							80.4 (22.6)
		Valid questionnaires							60

Abbreviation: SD, standard deviation.

Regarding the TAS, 51% of the patients scored 7 or above pre‐injury. Three years after the surgery, 73% of the patients returned to their pre‐injury level. After 10 years, 52% still performed at their pre‐injury level. During the follow‐up, 77% of the participants performed equally or at one level below their pre‐injury level, while 23% performed at two or more levels below.

**Figure 7:**
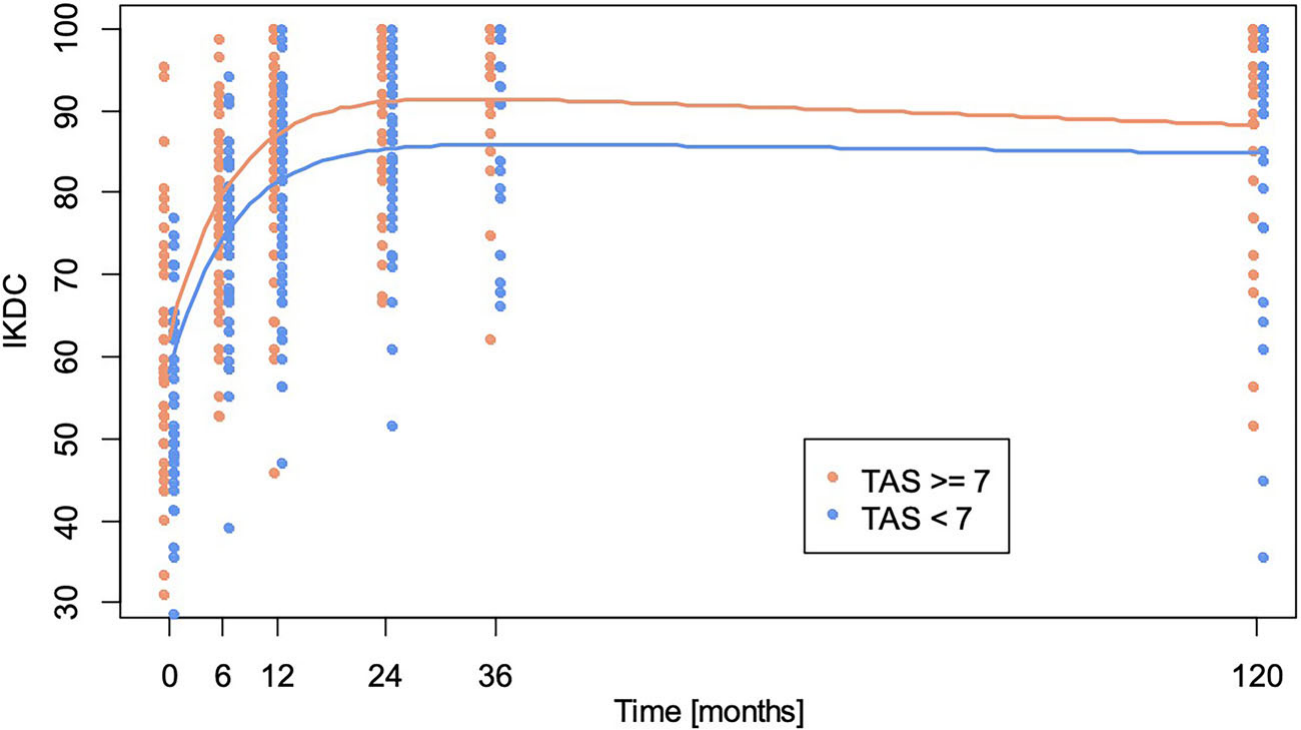
Results of the mixed‐model analysis on subjective knee function between competitive (TAS ≥ 7) versus non‐competitive (TAS < 7) athletes. IKDC, International Knee Documentation Committee; TAS, Tegner Activity Scale.

There was a significant difference (*p* = 0.0014) in the knee function (IKDC score) when comparing competitive athletes (TAS ≥ 7) with non‐competitive athletes (TAS < 7) over the 10‐year follow‐up period, indicating that elite athletes achieve better values. Furthermore, the analysis on patients of different genders revealed no significant difference (*p* = 0.3927) in the IKDC score between male and female patients during the follow‐up measurements. Figure 7 displays the individual data points and the fitted model curves.

#### RTS rates

Fifty‐three patients completed the questionnaire on RTS; the resulting data are presented in Table 3. On average, the participants returned to participation in sports within 5.9 months; 96% were able to return to their previous type of sport with a mean time of 9.6 months, while 72% returned to their pre‐injury type and level of sport within a mean of 12.2 months. Twenty‐six participants were involved in competitive sport (TAS ≥ 7); 65% of these were able to return to their pre‐injury level of performance.

**Table 3:** Different stages of return to sports and the time when they were achieved.

	Return to participation	Return to sports	Return to performance
Time in months (SD)^a^	5.9 (3.7)	9.6 (5.2)	12.2 (5.3)
Median (range)	5 (1–18)	8 (3–24)	12 (4–24)
Return (yes:no)	53:0	51:2	38:15
	100%:0%	96%:4%	72%:28%

a Values are given as a mean (standard deviation [SD]).

**Figure 8:**
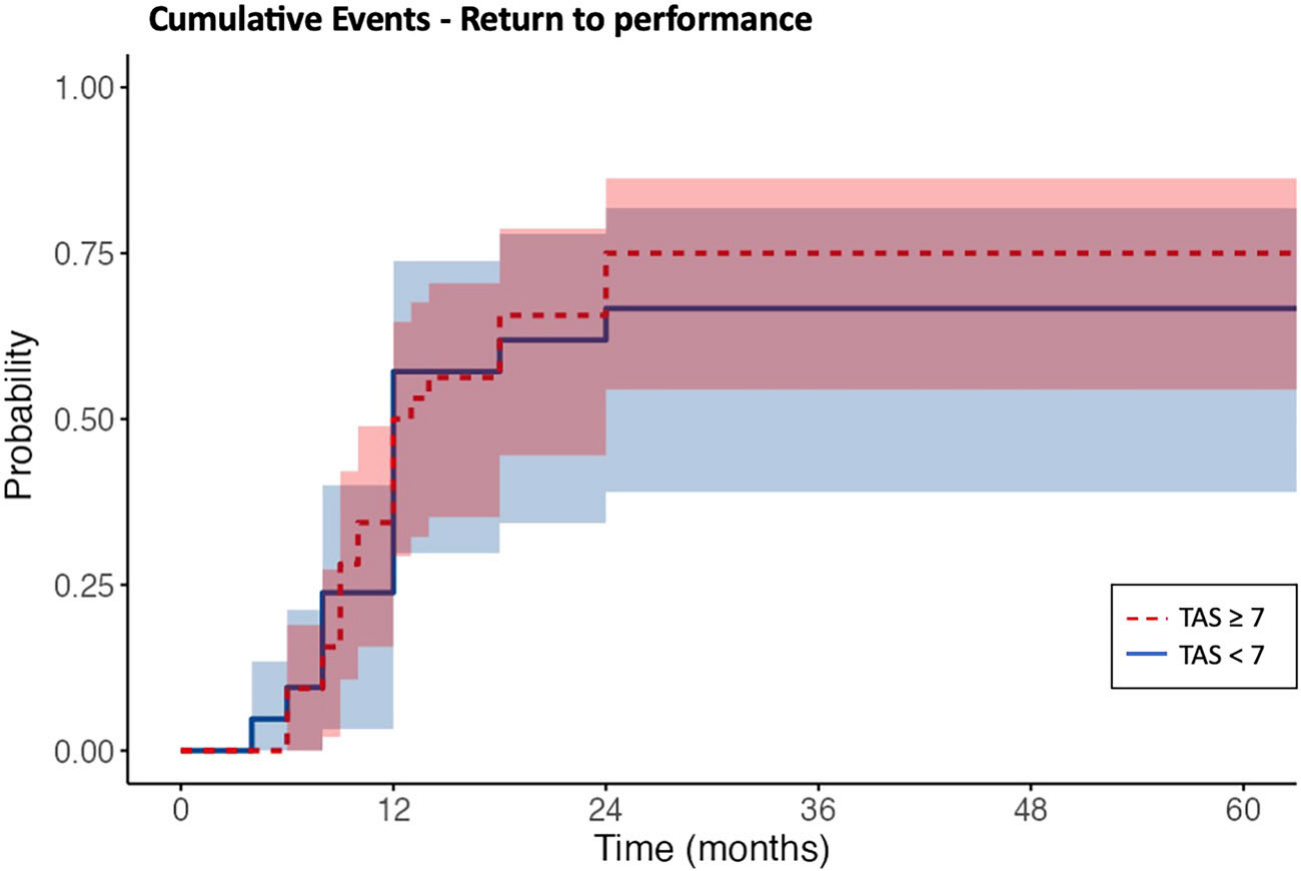
Time needed for RTP competitive athletes (TAS ≥ 7) versus non‐competitive athletes (TAS < 7). TAS, Tegner Activity Scale.

Figure 8 presents a comparison of the time required for successful return to pre‐injury performance between the competitive (TAS ≥ 7) and non‐competitive (TAS < 7) athletes. The log‐rank test was used for survival analysis and revealed no significant difference (*p* = 0.62) between these two groups. A gender‐based analysis of the time needed to return to performance successfully revealed no significant difference (*p* = 0.13).

#### Re‐rupture rates

All reported cases of ACL re‐rupture were recorded. Out of 281 patients identified as candidates for the study, a total of eight re‐ruptures were observed during the initial 3‐year follow‐up period resulting in a re‐rupture rate of 2.85%. All eight patients affected were men, with a mean age of 25.1 years at the time of surgery; six of these experienced a re‐rupture while participating in pivoting sports.

At the 10‐year follow‐up, 60 patients responded to our invitation to participate in the study; of these, 3 reported a re‐rupture, hence the re‐rupture rate between 3 and 10 years post‐surgery was determined to be 5.0%. Seven contralateral ACL ruptures were reported between 3 and 10 years post‐surgery. Of these 10 patients with re‐ruptures or contralateral ruptures, 7 were male; the mean TAS pre‐injury was 8.3, while the mean age at surgery was 20.8 years.

## DISCUSSION

After ACL reconstruction using the Press‐Fit‐Hybrid fixation technique, we found low re‐rupture rates, high rates of RTS and favourable patient‐reported outcomes, indicating that Press‐Fit‐Hybrid can restore functionality to the patient's knee, resulting in prolonged benefits and improved quality of life up to a 10‐year follow‐up period.

### RTS

The present analysis showed high rates of return to participation (100%), RTS (96%) and return to performance (72%). Regarding the TAS, 77% of the participants were able to perform their type of sport at the same or only one level below their pre‐injury level, while 65% were able to return to competitive sport at their pre‐injury level. No significant differences in the time to successful return to pre‐injury performance between competitive and non‐competitive athletes, or between male and female patients, were found.

A successful and safe RTS is a major goal of the ACL reconstruction itself and, in particular, the subsequent rehabilitation [25]. Systematic reviews analysing RTS after ACL reconstruction found pooled RTS rates ranging from 74% to 87% [3]/82% [4] for return to participation, 59% to 72% [3]/63% [4] for RTS and 46% to 63% [3]/44% [4] for return to performance. The review [3] indicated that 55% of patients were able to return to a competitive level of sports following their ACL reconstruction. Compared to these studies, the data found in our study indicates that Press‐Fit‐Hybrid enables comparably higher rates of successful RTS than in other reconstruction techniques, even in competitive athletes.

### Patient‐reported outcomes

Regarding the subjective functional outcome, the study found mean functional scores (Lysholm and IKDC scores) of 93.3 and 89.2 at the 3‐year follow‐up, and 90.6 and 86.5 at the 10‐year follow‐up. These values are comparable to the long‐term values presented by Barié et al. [5] (92 and 90 at 7.5 years) and Lee et al. [19] (89.7 and 90.9 at >7 years). In our study, good or very good outcomes were still demonstrated in 75.0% (IKDC) and 80.0% (Lysholm) of patients on the final follow‐up, which seems adequate when compared to Lee et al. [19] (77.3% [Lysholm]) and Barié et al. [5] (88% [IKDC] and 83% [Lysholm]). A systematic review [23] of studies with at least 5 years of follow‐up found good or very good outcomes in 48%–97% (IKDC) when a patellar‐tendon graft was used, and in 50%–97% (IKDC) when a hamstring graft was used. Leiter et al. [20] conducted a long‐term study on ACL reconstruction using hamstring tendons and bioscrew fixation, with a mean follow‐up of 14.6 years; they found a mean Lysholm score of 76.7 with 54.5% of patients reporting good or very good outcomes.

As recommended by Svantesson et al. [35], the study's focus on long‐term functional outcomes, patient satisfaction and knee‐related quality of life is addressed by the KOOS‐QoL. On the final follow‐up, a mean score of 80.4 was observed, with 70.0% scoring above 80. Compared to the normative score for their age group, 65% of our patients scored at or above the average level of patients without knee problems and, therefore, reported a similar quality of life regarding their knee [29]. In addition, only 7% of patients were aware of their knee problem daily, while 8% had a severe to extreme lack of confidence in their knee. Fifty‐two per cent reported no difficulty and 40% reported mild to moderate difficulty with their knee 10 years post‐surgery. A systematic review determined pooled rates of KOOS‐QoL from 9 studies with a mean follow‐up of 9 years after ACL reconstruction; this yielded a mean of 75 (95% confidence interval [CI]: 68.3–80.7) [11]. In a study designed to establish normative values for patients without ACL tears, meniscal injuries or osteoarthritis, the average score across all age groups was 82 (95% CI: 79.9–84.9) [11].

### Re‐ruptures

As the success rates of establishing mechanical stability through different graft choices and fixation methods have been widely discussed [6, 27, 31, 33, 37], the present evaluation focuses on graft stability by analysing re‐rupture rates. According to our data, the re‐rupture rate was 2.85% (*n* = 8 out of 281) within the first 3 years and 5% (*n* = 3 out of 60) between 3 and 10 years post‐surgery. The hospital specifically targeted therapy towards athletes, with 51% of the patients having a TAS ≥ 7 pre‐injury; 46% of the patients were under 30 years at the time of surgery, while 60% were male. In the present study, the majority of ACL re‐ruptures occurred in young male patients with high levels of sports activity; these factors were identified by Zhao et al. [41] to increase the risk of ACL re‐rupture.

When using the interference‐screw for fixation, re‐rupture rates have been found to be 5.8% [19] and 9.4%–11.1% [33] and 9% [5] when using a press‐fit technique with quadriceps‐tendon‐patellar‐bone. A systematic review on nine studies with at least 10 years of follow‐up found an overall risk of 7.9% [24] for re‐ruptures. Using a similar hybrid fixation method, Volz and Borchert [37] observed a re‐rupture rate of 4% after 3 years. In consideration of the low re‐rupture rates observed in our study, Press‐Fit‐Hybrid can be considered a mechanically stable and durable method for ACL reconstruction.

We discovered seven contralateral ACL ruptures in our final follow‐up, resulting in a rate of 11.7%, which is more than double the re‐rupture rate (5%). This is comparable to other long‐term studies, as a systematic review [24] found pooled rates of 12.5% for contralateral ACL ruptures among nine studies using different fixation methods with a follow‐up of at least 10 years. Our study confirms the review's findings that contralateral ruptures are more common than ipsilateral re‐ruptures [24].

The categorisation of re‐ruptures into two distinct rates may appear unconventional; however, this approach was deemed essential to ensure the accuracy of the data and information presented. The implementation and execution of the 3‐year re‐rupture rate adheres to prevailing publication standards [33, 37]. Nevertheless, due to the protracted follow‐up period, there was a loss of patients, which could potentially lead to a bias when calculating a single up to 10‐year re‐rupture rate. This is due to the fact that our knowledge of re‐ruptures that occurred in the period from 3 to 10 years post‐surgery is limited to those patients who had completed the final survey. Consequently, a second re‐rupture rate was calculated based on patients who participated in the 10‐year follow‐up, with the objective of facilitating a comparison of long‐term outcomes with those of other studies.

### Strengths and limitations

Only one fixation technique was used; thus, there was no control group. The results should be interpreted as preliminary and our findings should be validated using a randomised controlled approach. Moreover, data concerning further surgical interventions for meniscus issues subsequent to index surgery cannot be presented, as the questionnaires administered during the initial 3 years of the follow‐up period concentrated on outcome scores, and as the study's design did not include the evaluation or necessity of additional procedures following index surgery. The outcome measures employed in this study are of a subjective nature, as they are patient‐reported. Nevertheless, they are well validated, and the study design is consistent with that of other studies that have been conducted [9, 25, 31, 35, 38].

Furthermore, the data up to 3 years post‐surgery were analysed retrospectively; thus, there is the possibility of selection bias because only patients who had completed the questionnaire, at least at the 2‐ or 3‐year follow‐up, could be included in the study. For the 10‐year follow‐up, there may be a recruitment bias since 75 out of 135 patients either could not be contacted or did not respond to the invitation. The study design and the exceptionally long follow‐up period were responsible for a high rate of patient loss, which had the potential to bias the study's long‐term results. Nevertheless, when the present study is compared to similar studies analysing long‐term outcome after ACL reconstruction, the absolute number of patients participating in the final follow‐up is still adequate [5, 20, 39]. Furthermore, high drop‐out rates have also been reported [19, 20].

All surgeries were performed by the same surgeon; this could be interpreted as both a strength and a weakness.

This study is one of the first to report on the new Press‐Fit‐Hybrid technique and provides an extensive collection of data. It covers an exceptionally long follow‐up period and includes several measurement points in the first 3 years post‐surgery, allowing for a thorough analysis of patient development. The study examines a wide range of outcome measures, providing analysis of the surgery's results on various aspects.

## CONCLUSION

Press‐Fit‐Hybrid fixation demonstrates long‐lasting stability, even in combination with the above‐average RTS rates, although this may result in increased mechanical stress on the implant. The subjective outcome measures are comparable to those of well‐known fixation techniques [5, 19, 20], restoring functionality to the patient's knee [3] and resulting in prolonged benefits and improved quality of life [11]. Press‐Fit‐Hybrid may be considered a viable alternative to commonly used fixation methods for ACL reconstruction.

Further investigation could involve randomised controlled trials as short‐term studies to analyse the process of regeneration and compare it with different surgical techniques, as well as long‐term studies focusing on osteoarthritis and secondary joint damage.

## AUTHOR CONTRIBUTIONS

Conception and design, data acquisition, statistical analysis and main writing: Niko Schauer. Statistical analysis and internal review: Daniel Niederer and Rejane Golbach. Internal review: Martin Fischer, David A. Groneberg and Fabian Holzgreve. Conception and design, data acquisition and internal review: Daniela Ohlendorf and Adalbert Missalla.

## CONFLICT OF INTEREST STATEMENT

Adalbert Missalla has non‐financial interests as his patients were used for conducting the study. The remaining authors declare no conflicts of interest.

## ETHICS STATEMENT

The study was approved by the ethics committee of the Faculty of Medicine at the Goethe University Frankfurt am Main (No.: 2023‐1229). Written and oral informed consent was obtained before participating.

## Data Availability

The data that support the findings of this study are available from the corresponding author upon reasonable request.
